# Premastication—Review of an Infant Feeding Practice and Its Potential Impact on Allergy and Microbiome Development

**DOI:** 10.1111/all.16676

**Published:** 2025-09-08

**Authors:** Anne Steinberg, Sophie Goebel, Tara Eckert, Meral Sturmfels, Lara Meixner, Stefan Schülke, Katharina Blumchen, Kirsten Beyer, Birgit Ahrens

**Affiliations:** ^1^ Department of Paediatrics, Division of Pneumology, Allergology, Infectious Diseases and Gastroenterology Goethe University Frankfurt Frankfurt am Main Germany; ^2^ Department of Paediatric Respiratory Medicine, Immunology and Critical Care Medicine Charité – Universitätsmedizin Berlin, Corporate Member of Freie Universität Berlin and Humboldt‐Universität Zu Berlin Berlin Germany; ^3^ Paul‐Ehrlich‐Institut, Allergology Division Research Allergology Langen Germany; ^4^ German Center for Child and Adolescent Health (DZKJ) Partner Site Charité Universität Berlin Berlin Germany; ^5^ Paul‐Ehrlich‐Institut, Allergology Division Allergology Clinical Assessment Section Langen Germany

**Keywords:** environment and hygiene hypothesis, food allergy, microbiome, nutrition, pediatrics, prevention

## Abstract

Premastication, or pre‐chewing, of food as a feeding practice for infants has been practiced across cultures as an ancient evolutionary method. Whilst literature on the topic remains slim, the majority of existing research has highlighted the potential risks, such as transmission of infections. Although the concerns are valid, potential beneficial aspects have, until now, received less attention. These benefits are hypothesised to include exposure to a healthy, balanced oral microbiome, in combination with the anti‐inflammatory properties of saliva and the pre‐digestion of food by salivary enzymes. The hypothesis is supported by various studies that have shown the importance of early exposure to microbes for the development of the child's immune system. Moreover, a more varied microbiome earlier on in life is assumed to reduce the development of atopic diseases. Provided that the person chewing and its receiver/the child are healthy, premastication could offer a simple, well‐rehearsed method to shape the child's immune system with health‐promoting effects particularly in regard to primary allergy prevention. The interactive benefits of transferring an immune‐stimulating pre‐digested soft food portion containing small amounts of (diverse) food proteins via the oral route could be a valuable contribution to oral tolerance development in the decisive period of microbial‐driven immune system maturation. This review aims to evaluate the risks but especially the potential benefits of premastication, by focussing on its possible implications in (food) allergy prevention and oral tolerance development.

## 1 Introduction

Premastication, or pre‐chewing, of infant food refers to the practice of feeding an infant food that has been pre‐chewed by the caregiver or another adult, mostly in order to soften the food. It has been a common practice in human societies throughout the course of human evolution [1]. This practice is often performed during the weaning process, the phase between introducing solid food for the first time and reducing breastmilk or breastmilk substitutes. Premastication has been argued to help in supplementing the increasing nutritional needs of a growing infant, who has difficulty chewing solid foods due to the missing and still developing teeth.

So far, data on the occurrence, potential benefits, and risks of premastication are limited. Technological progress and the availability of commercial complementary foods may have replaced premastication and led to this method being largely unknown in Western societies. Nevertheless, the practice of premastication is seen in a vast number of cultures (see Table S1). Many potential benefits have been attributed to it [1, 2], such as strengthening the mother–child bond, supporting the development of the immune system or preventing choking hazards for small children by softening the food. However, most of the literature tends to focus on the risks and dangers of this practice [3, 4]. These include the transmission of infectious pathogens like human immunodeficiency virus (HIV) [5, 6, 7, 8, 9] or *Streptococcus mutans* causing early childhood caries [10, 11, 12]. However, the transmission of saliva and microbes from a healthy donor could be instrumental in the evaluation of premastication (see Table 1).

**TABLE 1:** Summary of potentially positive and negative aspects of premastication based on publications shown in Figure 1 and Table S1 considering nutritional, microbial, immunological, and general aspects.

Premastication	Potentially positive	Potentially negative
Nutrition	Greater variety of (tough) foods and nutrients can be consumed by being able to eat food otherwise not mechanically possible due to the not fully developed chewing apparatus of the infant [1]	
	Salivation facilitates food gliding, reducing choking hazards and cooling/warming food for child [1, 2]	
	Adding amylases via saliva aids digestion [1, 16, 17]	
Microbial contact	(Enhanced) early life contact with various (oral) microbes or microbial components [18, 19, 20] Helping shape microbiome development and maturation Helping shape the formation of neutralising antibody isotypes	Transfer of (oral) bacteria that can be pathogenic for example causing early childhood caries [10, 11, 12], syphilis [21, 22, 23], or stomach ulcers [24]; Ref Table S1: [12, 55, 56]
		Transfer of viruses (including HIV, HBV) via saliva [5, 6, 7, 8, 9, 27, 28, 29]
Immune system	Transfer of cytokines and immunoglobulins via saliva [16] Immunomodulating effects promoting mucosal immunity and oral food tolerance	
	Microbiome development (Encounters with microbes and microbial components) interacting with immunomaturation, long‐term programming of immune cells [13, 14, 30]	
Others	Emotional bonding [1]	Considered socially “unfavourable” behaviour in some countries [1]
	Enhanced practicability (no need for “external” equipment for chopping/cooking/softening the food) [1]	
	Food from the “family table” [31, 32]	

Indeed, in further development of the hygiene hypothesis, the avoidance, not the exposure to microbes and microbial components, is assumed to contribute to the increase in atopic diseases [18, 33]. Over the last decade, evidence for the general impact of early microbial inputs, such as bacteria‐derived compounds and metabolites (e.g., lipopolysaccharides (LPS), flagellin) or viral pathogen‐associated molecular patterns (PAMPs), on the development of the child's immune system has been growing [34]. Hence, various factors such as the mother's microbiota, birth mode, breastfeeding, dietary exposures, the number of siblings or contact to animals have been highlighted to shape the early‐life colonisation in a beneficial manner [18, 19]. Indeed, disparities in the gut microbiota have been associated with atopic diseases, such as a delayed gut microbiota maturation in the first year of life being “a hallmark of paediatric allergic disease,” [35] or a low gut microbiota diversity in early infancy as a possible predictor of asthma at school age [14, 36].

**FIGURE 1:**
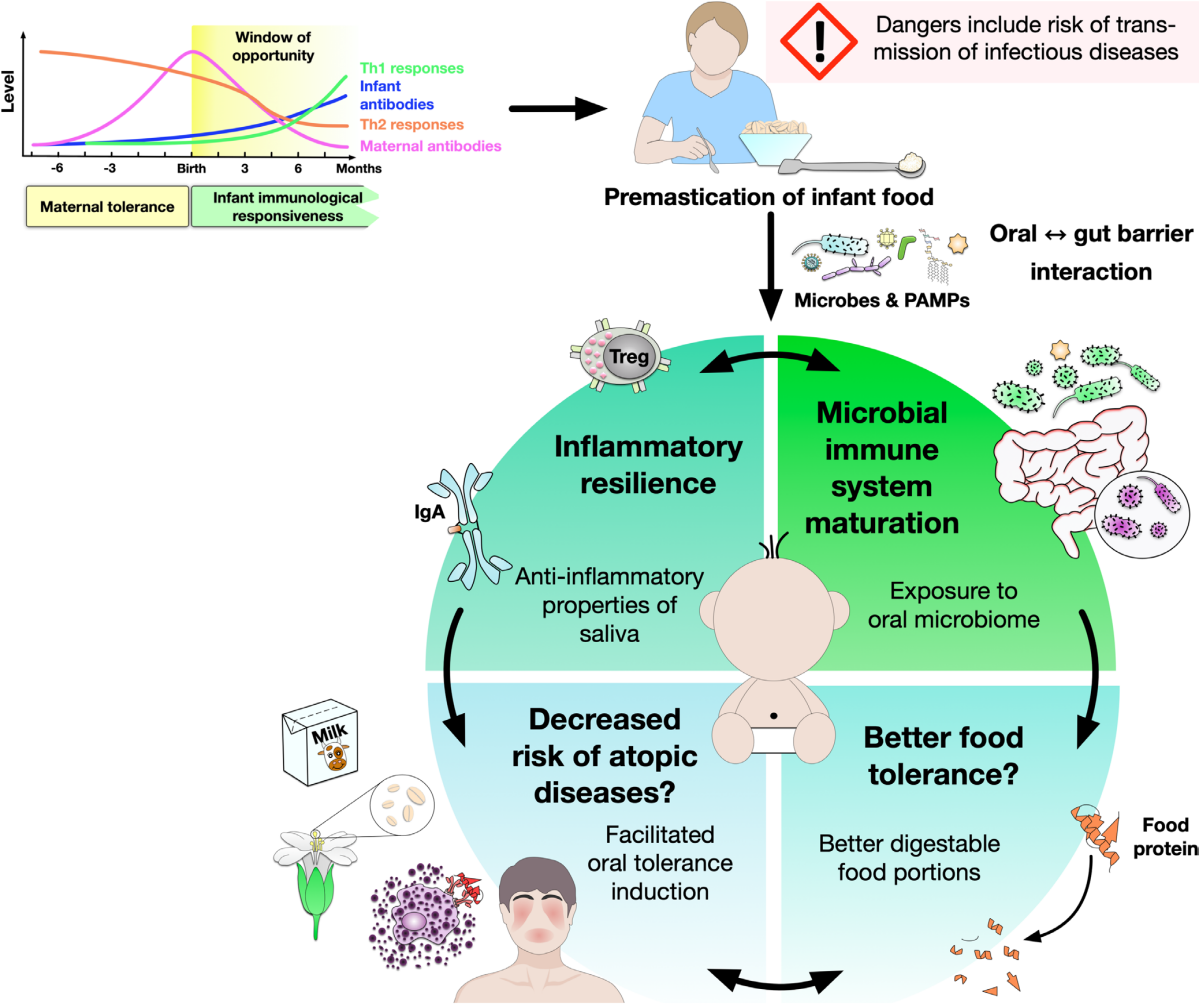
In the infant‘s most formative time window [13] the interplay of increased (first) contacts to the outer world via premasticated food is assumed to additionally stimulate microbial‐driven immune system maturation and inflammatory resilience. Microbial colonisation and subsequent immune maturation depends decisively on microbial contacts. Th1 cell‐mediated responses to the colonising (gut) microbiota are supposed to extenuate potentially pathologic Th2 cell‐mediated responses [14], underlining the importance of the Th1‐Th2 balance for immune‐priming. Introduction of the soft, insalivated, and pre‐digested premasticated food some months after birth in turn is assumed to support nutrient absorption and promote oral food tolerance [14]. Together, these factors are suggested to create an anti‐allergic milieu and thus favor a decreased risk for atopic diseases [15].

Likewise in saliva, a lower diversity of oral bacteria was detected in allergic children [37]. According to Kunath et al. [38], the oral cavity is suggested to act as a gateway for microbial colonisation, also shaping subsequent microbial communities in the digestive or respiratory system, but knowledge is limited [38, 39, 40]. Here, the dynamic interplay between the oral cavity and the gut, two anatomically continuous regions with rather differently equipped microbiomes, separated by the (mature) oral‐gut barrier with its various “lines of defence” (described as the physico‐chemical, the immune and the biological barrier, of which saliva is particularly important), seems to play a decisive role [38]. The (mucosal) immune system is assumed to be “activated” in parts through first contacts with foreign proteins, including pathogens as well as food proteins [38]. In correspondence, the balance of immunological reactions induced by these interacting stimuli may represent one central part of the infant's immune system maturation [41]. Food allergies typically have their first manifestation in early infancy. According to the information presented above, the premasticated weaning food, including the targeted confrontation with the donor's oral microbiome and the anti‐inflammatory properties of its saliva, could be seen as an opportunity for a healthy immune imprinting for the growing child, especially in terms of oral tolerance acquisition [13] (see Figure 1).

**FIGURE 2:**
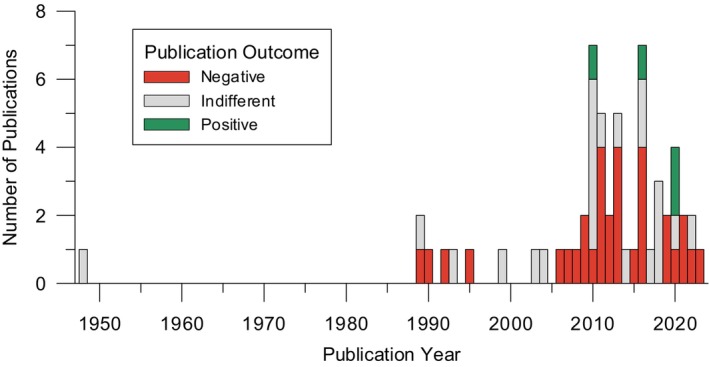
Graph showing the number of publications appearing on PubMed when using the search terms: “premastication OR pre‐mastication OR prechewing OR pre‐chewing”, arranged by year and view of premastication (According to the view on the feeding practice, we divided the publications into three “outcome categories”: Positive (predominantly beneficial, green), negative (predominantly voicing safety concerns, red), or indifferent/mixed (cannot be clearly assigned to positive or negative, grey)). Twelve publications were not directly related to the feeding technique and are therefore not included. The number of publications remains small, but a distinct growth in publications is visible from 2010 onwards. The focus of the publications is clearly on the negative outcomes of premastication. Disclaimer: The information presented in this figure reflects solely the personal opinions of the authors of this review.

To our knowledge, there is no current review which analysed the existing literature on the potential risks and benefits of premastication with a special interest in influencing the microbiome of the infant and thereby atopic disease prevention. In general, only a handful of studies have been published in the field of premastication, with the majority being either case reports or epidemiological findings as part of a retrospective cohort study (see Table S1). When performing a PubMed search (see Methods), it is of note that only 68 search results were retrieved within the last 76 years, further highlighting the lack of research on the topic of premastication. However, it appears that there is a growing number of studies in recent years (see Figure 2) which could be argued to correlate with a growing interest in infant feeding practices and ”hygiene” likely due to Pelto et al.'s [1] first anthropological review on the topic.

**FIGURE 3:**
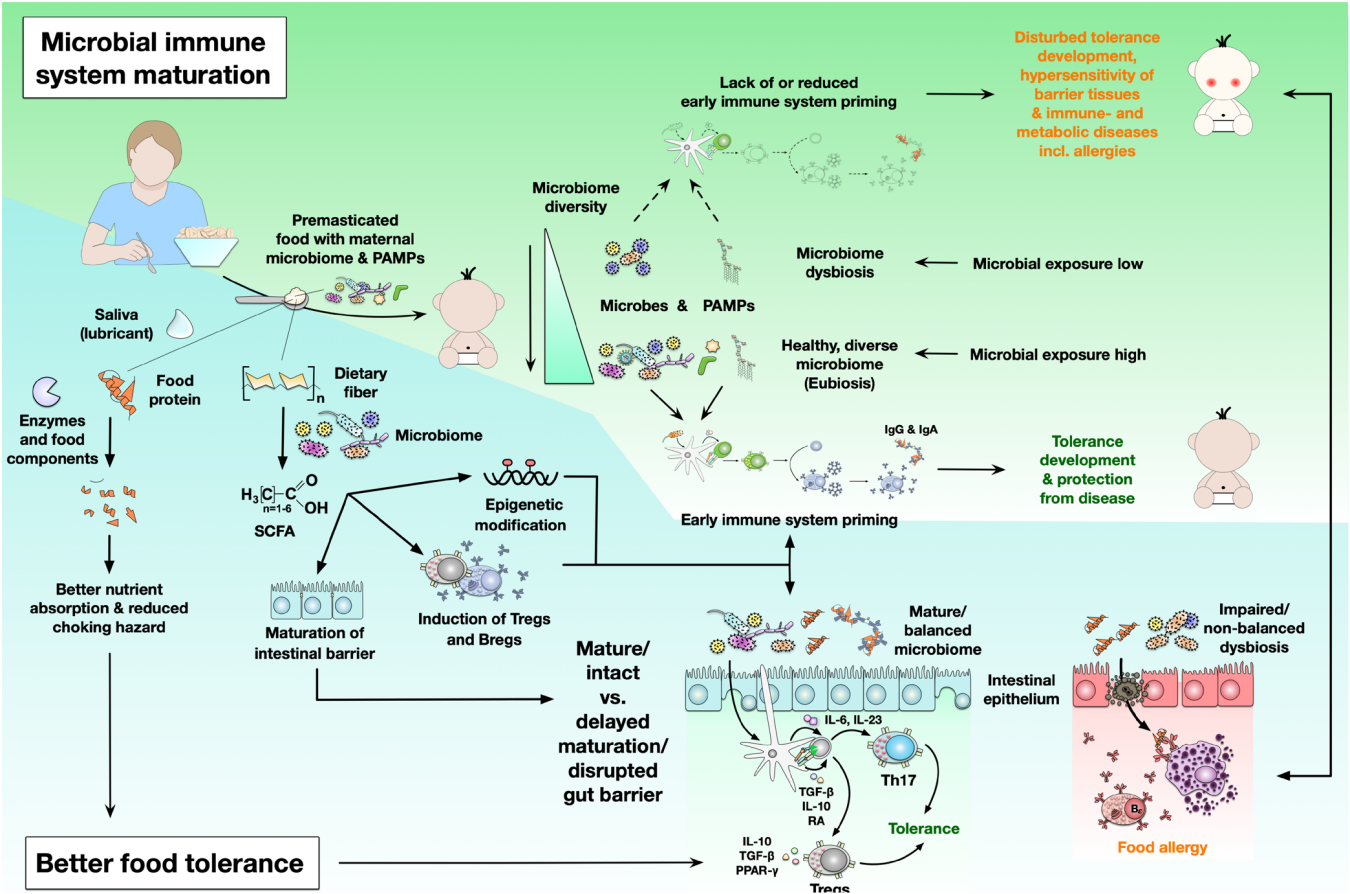
Hypothetical effects of premastication on “Microbial immune system maturation” and “Better food tolerance”: Increased exposure to microbes or microbial compounds is regarded as essential for normal immune maturation [13]. By transferring the oral microbiome of the prechewer, microbial inputs may be further increased. The premasticated soft and insalivated food is assumed to support nutrient absorption and oral tolerance [15]. Containing prechewd fibres of “tough foods” are assumed to support maturation of epithelial barriers, as well as their maintenance e.g., by production of short‐chain fatty acids (SCFA) and other metobolites [42, 43, 44]. In summary, oral food tolerance development, with participation of e.g., inducing transforming growth factor‐β (TGF‐β)‐, and interleukin‐10 (IL‐10)‐producing regulatory T cells is assumed to be promoted.

**FIGURE 4:**
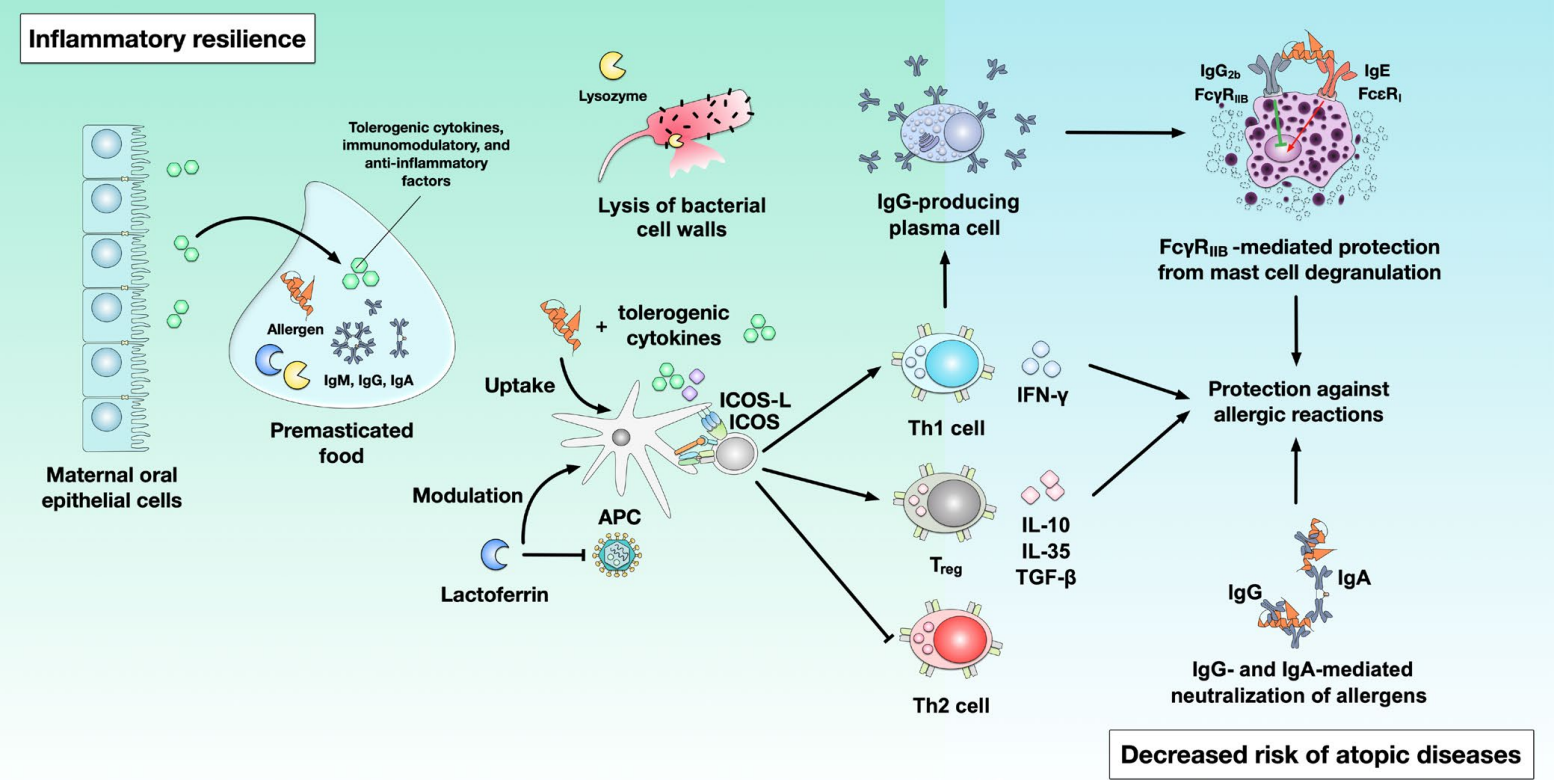
“Inflammatory resilience” and “Decreased risk of atopic diseases”: Taking place in the mouth and digestive tract of the infant, maternal saliva with its antiviral, immunomodulatory, and anti‐inflammatory properties, including Lactoferrin [45] or Lysozyme [46, 47] as well as immunoglobulins (Ig) such as IgG, IgM, and IgA is mixed with the premasticated food chyme and passively transferred to the child (in it‘s period of fading maternal passive immunity). These factors are supposed to support an immunologically favourable milieu [15]. Early inducible T‐cell co‐stimulator (ICOS)–mediated T cell activation promotes the differentiation of antigen‐specific Th1‐ and regulatory T cell (Treg) subsets while suppressing Th2‐differentiation [48]. Subsequently, an anti‐allergic milieu is supposed to be promoted, characterised by the secretion of typical tolerogenic cytokines such as IL‐10, IL‐35, or TGF‐ß, or the induction of Treg subsets, which may prevent the development of atopic diseases.

In this review, existing literature will be evaluated with regard to epidemiological information as well as recent findings, aiming to complement Pelto et al.'s [1] review. We will further highlight the potential influences on the microbiome of the infant and illustrate our mechanistic considerations on its implications in the maturation of the immune system, especially in the context of atopic diseases and oral tolerance development (Figures 1, 3 and 4). We hereby want to provide a new approach to evaluating the benefits of premastication.

## 2 Methods

In this review, we screened all results from a PubMed search in JUN 2024 using the search terms “premastication OR pre‐mastication OR prechewing OR pre‐chewing”. In total, only 68 results were retrieved, dated between 1948 and 2024 (see Table S1). Twelve of the 68 papers were not directly related to the feeding technique, as these addressed for example dental health, neuroscientific effects of gum chewing or swallowing impairments. We evaluated the remaining results regarding information on occurrence, including epidemiological information, potential risks and benefits and general attitude towards premastication. In relation to the latter, we categorised the publications as „positive“ (predominantly beneficial effects associated with premastication), „negative“ (predominantly voicing safety concerns), „indifferent“ (neither positive nor negatively associated) or “mixed” (beneficial effects associated with premastication but also stated safety concerns) based on their association with the feeding practice (see Figure 2). Additionally, we included publications on the development of the microbiome and/or allergies in children in this review to evaluate not yet discussed benefits of premastication. We have summarised those publications including their particularly important findings in a separate table (see Table S2).

## 3 Cultural Presence

Although the historical and anthropological literature on premastication is not extensive, a number of reports suggest premastication to be an ancient practice that was undertaken across cultures. Earliest accounts date back to 1500 years ago in ancient Egypt where the Ebers medical papyrus mentions a mother spitting “a remedy” into a child's mouth [49]. Further, Ibn Sina (Avicenna) “The Prince Of Physicians” (980–1037 ad) from present day Uzbekistan [50] mentioned in his five‐part Canon of Medicine “Weaning must not be abrupt. After the first two teeth have appeared, introduction of solid foods is to be considered. At the beginning, premasticated bread should be given…” [51].

Whilst there is little epidemiological data, premastication can still be observed across cultures today. For instance, Pelto et al. [1] used the Human Relations Area Files online data base (e‐HRAF) for a cross‐cultural examination of the prevalence of premastication. The ethnographic data base provided information about weaning in 119 of 155 cultures. Premastication was mentioned in 38 cultures, most frequently described in Asian cultures. However, the impact of the data is limited, as it is based on ethnographic observations and primarily describes social and cultural features. Pelto et al. [1] therefore discuss whether premastication is underreported. In a second analysis, they found that 65 of 104 families (63%) from the Han Societies in China had practiced premastication. More recently, Czarnik et al. [52] reported instances of premastication in the longitudinal Infant and Toddler Feeding Practices Study‐2 (ITFPS‐2) by the US Department of Agriculture's Special Supplemental Nutrition Program for Women, Infants and Children (WIC). Recruitment for the study took place in 2013. More than 1900 participants completed the survey at four survey time points (at infants age 7, 9, 11 and 13 months) (*n* = 1904). 11.6%–17.7% claimed using premastication as a form of feeding their infant between the ages 7 to 13 months. There were significant differences between the included ethnicities regarding different food preparation practices, also including performance of premastication: Whilst non‐Hispanic White and Hispanic individuals prechewed less, non‐Hispanic Black caregivers practiced premastication more frequently. In month 11, 42.5%, 8.9% and 11.6% of non‐Hispanic Black, non‐Hispanic White and Hispanic caregivers reported pre‐chewing, respectively. Other reported examples of premastication are derived from indigenous Tsimane tribes of the Bolivian Amazon [2], areas in South Africa [7], and in Laos [53], making it a commonality across different cultures.

Several socio‐economic factors have been shown to be associated with premastication. According to Zhao et al. [54], mothers from a lower academic background in China are more likely to practice premastication. Further, Gaur et al. [55] reported that the awareness of pre‐chewing infant food increased with lower education levels. Furthermore, age, availability of electricity and the number of household members had a significant association with pre‐chewing infant food, with women of older ages and living with fewer relatives, as well as those with access to electricity, being more likely to practice premastication [56]. Hafeez et al. [25] also state that mothers who experienced premastication as a child are seven times likelier to continue the practice with their children. The latter might highlight a cultural importance of premastication.

## 4 Reasons for Practicing Premastication

One reason for premastication is mechanical in nature, bridging the mismatch between child (teeth) development and growing nutritional needs [1]. As a whole, the development of the “mastication apparatus”, including the bones (maxilla and mandible), muscles (including tongue control) as well as teeth/teeth eruption (the central incisors erupt between 8 and 12 months of age; the lateral incisors erupt between 9 and 18 months) and soft tissue, influence the ability to chew and the stage of the chewing patterns [26]. Mastication kinematics over time show that “at 4–6 months of age, jaw movements are simple elevations, assisted by actions of the lips and tongue. The next stage in the development of chewing is marked by the emergence of lateral jaw motion to finally reach a rotary jaw movement, which is the sign of mature mastication at the age of 24–30 months.” [26] In accordance to this, Pelto et al. [1] argue that premastication was an important mechanism to bridge the gap between the halting of breastfeeding and the development of teeth ‐ and correspondingly of mature chewing patterns in infants. The authors argue that it allowed infants to consume a great variety of nutrients that they would otherwise not be able to eat due to the delayed tooth development in humans compared to other mammals, thereby preventing malnutrition and giving humans an evolutionary advantage. In a survey on the topic, Pelto et al. [1] further describe that “the most frequently premasticated food was meat (authors' note: providing an iron supply), followed by rice, other grains, other ‘tough foods’ and nuts.” [1] Additionally, the act of chewing adds saliva to food, which acts as a lubricant for the digestive tract, but is also important in adding amylases to foods which aids digestion and nutrient absorption [16]. Further reasons for premastication include allowing the uptake of food that is otherwise too hot or too dry or poses a choking hazard for infants [2] (Figure 3).

Using the e‐HRAF database, Pelto et al. [1] found that the primary reason for premastication was to provide food (31 out of 38 cultures). However, participants from four of the cultures that practice premastication said that they do so for a beneficial outcome that is rooted in cultural or religious beliefs. For instance, premasticated herbs were used medically to cure diseases [1]. The notion of premasticated food being used in a cultural or traditional setting can be seen in the Islamic tradition of Tahnik (or Tahneeq), which is a ceremony in which the palate of a newborn baby is rubbed with honey, premasticated dates or a sweet juice [57], with references of this dating back to 870 ad “so that he should rub his palate with chewed date.” [58] Interestingly, research suggests that this practice may have immunomodulating effects in the sense of an increased expression of interleukin (IL)‐12 (IL‐12) in the palatal and gingival mucosa. Increased expression of IL‐12 and an increase in CD8^+^T lymphocytes in comparison to the control group was determined by using immunohistochemical staining and flow cytometry of neonatal rats' blood, which received Tahneeq on the palatal and gingival mucosa immediately after birth [57]. Both markers represent important factors in the immune response, further adding to the idea of medicinal benefits caused by premastication.

## 5 Risks

The majority of publications on premastication to date have largely focused on the negative aspects of it (see Figure 2 and Table 1). Furthermore, the published literature suggests a lower prevalence of premastication in Western cultures compared to Asian or African (Table S1). Pelto et al. [1] argue that this occurred due to increased access to modern food processing and hygiene concepts that label premastication as unhygienic and dangerous.

Indeed, passing on oral bacteria, and thereby infections caused by pathogenic bacteria, is a valid concern. Imong et al. [59] found that, when analyzing the bacterial contamination of rice as a weaning food for infants, the total bacterial count of premasticated rice was significantly higher than that for non‐premasticated rice. This could be potentially dangerous when certain pathogenic bacteria such as *Streptococcus mutans* in the case of early childhood caries [10, 11, 12], *Treponema pallidum* in the case of syphilis [21, 22, 23] or *Helicobacter pylori* which is known to cause stomach ulcers [24], are included in the salivary bacteria. The transmission of these pathogens has been associated with premastication [10, 11, 12, 21, 22, 23, 24].

However, other research suggests a caries‐protective effect of a diverse, balanced oral microbiome as part of an oral ecosystem which needs biodiversity to respond adequately to fluctuations of environmental factors [30, 60]. Additionally, an infection with bacteria such as *H. pylori* does not necessarily result in pathological effects [61]. In fact, experiments in mouse models have shown an infection with *H. pylori* to have protective effects on asthma development, with mice that were infected early in life showing an increased induction of regulatory T‐cells (Tregs) and protection from the key aspects of asthma (airway hyperresponsiveness, tissue inflammation, and goblet cell metaplasia) [62].

Also, viruses can be passed on via saliva to infants, for example, through open wounds [63], via premastication or via utensil sharing. Crabtree et al. [27] showed in a prospective cohort of 270 Zambian children that sharing utensils with a primary caregiver is a risk factor for human herpesvirus‐8 (HHV‐8) transmission. Similarly, studies have shown that sharing food increases the risk of hepatitis B virus (HBV) transmission [28, 29].

Concerningly, a number of studies have shown an association between the transmission of HIV and premastication mostly due to mouth sores or bleeding gums as a cause for blood transmission via saliva [5, 6, 7, 8, 9, 55, 64]. According to a recent review of the literature on horizontal HIV transmission, premastication per se is regarded as unlikely to be the risk factor in horizontal HIV transmission but rather the exchange of bodily fluids (albeit through saliva or breastmilk) whilst a mother has a high viral load of HIV [8]. Baron et al. [65] found that saliva disrupts HIV‐infected mononuclear leukocytes. They argue that hypotonic disruption via the saliva could be a main mechanism by which the attachment of infected leukocytes to mucosal epithelial cells is prevented, thereby inhibiting oral HIV transmission. They argue that HIV transmission is higher in fluids such as milk and seminal fluids because saliva (as critical part of the oral‐gut barrier [38]) provides protective effects through this hypotonic lysis [65].

## 6 Benefits and the Microbiome

When focusing on the benefits of premastication, there are socio‐cultural as well as biological aspects to consider. Via informal interviews with mothers, Pelto et al. [1] found that some mothers see premastication as a way of reinforcing a special bond or “sharing love” with their infant. The nutritional benefits include not just preventing malnutrition in infants, by introducing proteins and other nutrients such as iron that would not be met by breastmilk alone [1, 66], but also strengthening epithelial barriers in the gut. The example of iron intake via pre‐chewed meat illustrates this point very well. Reported premasticated “‘tough foods’ and nuts” [1] suggest an (early) transfer of additional dietary fibre. Gut microbiota ferments dietary fibre into short‐chain fatty acids (SCFAs), which are regarded as major players to modulate immune homeostasis [42]. Both, dietary fibre and SCFAs are reported to enhance epithelial integrity [42], which might in addition support the maturation of the infant's epithelial barriers (Figure 3). Moreover, butyrate, one of the SCFAs, has been reported to regulate type 2 immunity [43]. In turn, dysfunctional microbiota with reduced capacity to produce butyrate have been reported as a basis for allergic disease [67]. The serving of pre‐chewed nuts may not only be rich in protein, fibre, and unsaturated fatty acids but also has its own charm from an allergy‐preventive point of view as being in line with current suggestions on the promotion of “early introduction” of allergenic foods for allergy prevention [68].

Another benefit of premastication may be the support in digestion and absorption of foods via the additional saliva of the prechewer [16]. The role of human saliva and its components is multifaceted, including the gastrointestinal functions of taste, chewing, enzymatic digestion—especially of starch and fats ‐ and swallowing (Figure 4) [17].

Saliva, much like breast milk, contains not only electrolytes, proteins, mucins and enzymes but also growth factors, antimicrobial factors and anti‐inflammatory factors which aid the body's repair mechanisms after tissue injury [16]. Among these is lactoferrin [45], which should be emphasised due to its antiviral, immunomodulatory and anti‐inflammatory properties as well as lysozyme [46, 47], an enzyme that can hydrolyse bacterial cell wall bonds. Importantly, saliva also contains immunoglobulins (Ig) such as IgG, IgM and IgA. These are essential factors that modulate the immune response [69]. Lack and Panagos argue in the Maternal Child and Nutrition commentary [15] to Pelto et al. [1] that saliva contained in prechewed foods could passively transfer these immunoglobulins and cytokines, creating an immunologically favourable milieu which both promotes oral tolerance and could play a role in preventing allergies (Figure 4).

The oral cavity is an immunologically relevant site where the immune system establishes first contact with pathogenic organisms and new antigens. Especially, the introduction of complementary foods in the weaning process leads to the presentation of many new antigens to the infant's immune system. Antigens are transported into the lymphatic system and presented by dendritic cells, macrophages, and B cells. The presence of certain mediators is considered to determine if the antigen is tolerated or not. For instance, following the inducible T cell co‐stimulator (ICOS) in its assumed role in directing effector T cell differentiation [48], the abundance of Tregs and levels of the anti‐inflammatory cytokine IL‐10 are associated with tolerance [47]. Thus, the oral mucosa, the oral epithelial cells (OECs), the associated secretory and lymphoid structures, including immunoglobulin‐producing plasma cells in the B‐cell compartment [70], and the secretion of IL‐10 together with transforming growth factor (TGF)‐β and IL‐35 by Tregs [71], may actively contribute to various immunoregulatory processes [47]. Therefore, one may argue that released cytokines and immunomodulatory factors secreted by maternal OECs may trigger protective and tolerogenic processes in the infant through the transfer of intensively salivated and pre‐chewed food portions. The transfer of these “immune‐stimulating” portions may be an important factor in the “Lack Dual Allergen Exposure Hypothesis”, which emphasises the early consumption of food protein for the induction of the infant's oral tolerance development [15, 72]. Even if the underlying pathomechanisms are not yet fully understood, premastication could be an additional piece in the puzzle to promote prevention of (especially food) allergies.

Microbiome studies have shown that bacterial communities in early life are vital in priming and “guiding” the development of the immune system and that the disruption of the child's microbiota correlates with immune and metabolic conditions [73]. In the context of the “evolved” hygiene hypothesis, which states that the emergence of allergies in the twentieth century could be due to a decrease of microbial inputs, “exposure, not avoidance, is what contributes to a child developing a healthy immune system.” [18] This strongly suggests that the microbiome could have a protective effect against some diseases. Another much‐discussed topic in terms of early‐life microbial exposure is the health‐promoting effect of vaginal delivery for the development of the child's immune system as well as the possibility of the procedure of “vaginal seeding” for unavoidable caesarean deliveries [74]. Furthermore, the importance of exposures to microbes and microbial components may primarily be placed during an early lifetime window of opportunity [14]. If missed, “pathological imprinting, altering risk for disease throughout life” might be the consequence [13].

It is argued that insufficient microbial stimulation during infancy may lead to the hypersensitivity of barrier tissues, which in turn increases allergy development [75]. In this line of thought, a number of early “sanitary” environments (such as an increased use of soaps and detergents, antibiotic usage as well as living in an urban setting) have been shown to increase the risk of atopic diseases [76]. A study found that respiratory allergies (such as allergic asthma and allergic rhinitis) were less frequent in adults heavily exposed to orofecal and foodborne microbes [77]. Again, this supports the hygiene hypothesis, particularly emphasising the importance of the early life exposure, when the microbiome is being developed and primed [13, 73].

Currently, research on the development and composition of the oral microbiome in early life is still limited. The composition of oral microbes in breast‐fed infants differs from formula‐fed infants [78]. The authors hypothesised that bacteria from breast milk colonise the infant oral cavity and that non‐microbial components influence the infant oral microbiome. The introduction of solid food and primary tooth eruption further contributes to changing the infant microbiome by providing new surfaces for bacterial attachment [78]. In this context, it is likely that the passing on of pre‐chewed food with bacteria from the caregiver's oral microbiome could influence the development of the infant's oral microbiome. Indeed, preliminary studies indicate an inverse association between allergies and the number and diversity of bacteria in the infant saliva or contact with parental saliva [20, 79].

Research has shown the importance of the gut microbiome in the pathogenesis of a vast number of diseases (including intestinal conditions, systemic diseases such as obesity and cardiovascular disease but also neurological diseases such as Parkinson's) [80]. The development of the gut microbiome, particularly the microbial colonisation in early life, has also been shown to be vital in reducing risk for disease in adult life, for example, type 1 diabetes, intestinal inflammation and obesity [13, 30, 73], and to be critical for the regulation of immune system maturation and allergy development in children [37]. Chaudhary et al.'s [81] recent review further highlights the interplay between microbiomes throughout the body in health and diseases and argue that by understanding the interplay and “leveraging the therapeutic potential of the gut microbiota” we can develop targeted interventions that prevent/treat diseases.

In fact, Feehley et al. [82] have shown in a mouse model that germ‐free (GF) mice, which had been colonised with bacteria from the faeces of healthy infants and subsequently sensitised with cow's milk protein beta‐lactoglobulin (BLG), were protected from an anaphylactic response to BLG challenge, but not GF mice colonised with bacteria from the faeces of cow's milk allergic infants and subsequent sensitisation and challenge with BLG. The authors argue that “intestinal bacteria are critical for regulating allergic responses to dietary antigens” [82], and that interventions that target the modulation of the microbiome could be relevant in treating food allergies.

The interplay between the oral and the intestinal microbiota especially in terms of (initial) colonisation have not yet been fully elucidated [38]. Based on the (mature) oral‐gut barrier, oral microorganisms may rather reach and colonise the gastrointestinal tract in the context of gut dysbiosis. The oral‐gut barrier with its “three main lines of defence” [38] (the physico‐chemical, the immune, and the biological barrier, of which saliva is a critical part) is thought to “prevent both the entry and translocation” (in the meaning of crossing the epithelial barrier) of oral microorganisms and pathogens to the gut [38].

In infants, the digestive system still needs to develop. Not only the microbiome but also differences in intestinal digestion and absorption functionality or motility may play a role. For example, the infants' gastric secretory capacity has been described to be below adult level and the postprandial gastric pH of infants is relatively high compared to adults [83, 84, 85]. Pancreatic amylase, important for carbohydrate hydrolysis, is described not reaching adult levels until about 2 years of age [86]. These and further continuous intrinsic developmental events may further influence colonisation milieu or epithelial permeability in response to exogenous stimulation. Recently, Ohtsuka et al. [41] discussed the immaturity of gut functions, including digestion and the (leaky) barrier protection against foreign molecules, highlighting its close relationship to the induction of tolerance. Tolerance, as an acquired mechanism of the (gut's) immune system, helps prevent excessive reactions to foods. Here, tolerance is mediated by, for example, TGF‐β‐, IL‐4‐ and IL‐10‐producing Tregs and stimulated IgA synthesis [41]. In this context, secretory IgA, with its assumed ability for muco‐trapping (clumping of bacteria) also regulates immune responses to dietary antigens in conjunction with neutralising and immune‐modulating properties of IgG (Figure 4) [41, 87].

Dzidic et al. [37] tested the theory of microbial colonisation via saliva and its influence on allergy development in children. They analysed data from longitudinal salivary samples of 80 infants (47 of which were developing allergy symptoms and 33 of which were healthy) was collected between 2001 and 2003 at 3, 6, 12, 24 months and 7 years of age. Bacterial load in the saliva samples was measured using quantitative polymerase chain reaction (PCR). They found that children developing allergic symptoms (particularly asthma) had a lower diversity in the salivary bacteria but also a divergent bacterial composition at 7 years of age compared to the healthy control group. The results indicate that these early changes in oral microbial composition, likely a consequence of an impaired immune system, influence immune maturation and allergy development. Additionally, it seems to suggest that acquiring a higher variety of microbes early on in life could prevent atopic diseases. Soriano et al.'s [79] findings that antiseptic cleaning of a pacifier could lead to a higher risk of a subsequent food allergy in children, as well as Hesselmar et al.'s [20] and Kubo et al.'s [88] results that parental sucking of their infants pacifier may reduce the risk of allergy development “possibly via immune stimulation by microbes transferred via the parent's saliva,” [20] further support the notion that premastication and saliva could be beneficial in passing on the “good bacteria and their components”, thereby aiding the development of the immune system and preventing atopic diseases.

To the authors knowledge, only two studies have investigated the direct link between premastication and microbiome transfer and/or allergy development. One of the studies is only referenced by the same authors in a later paper (Kubo & Yoshizawa 2015, cited by Kubo et al. 2023) [88], and could not be found when searching PubMed or Google Scholar as it appears to have only been published in Japan. Kubo et al. [88] argued that the earlier study showed that premastication during infancy reduces the risk of allergy development, particularly eczema, at school age, likely due to the transmission of oral microbes via saliva. Kubo et al. [88] themselves found that Japanese children who shared eating utensils during infancy with their parents were significantly less likely to have eczema currently (at an average age of 9–13 years). Surprisingly, in the second study found on this topic, Han et al. [2] show that when extracting bacterial DNA from 2 premasticated food samples and 12 matched salivary samples from maternal‐infant pairs of Tsimane mothers, salivary microbiota in infants does not appear to be closely related to their mothers' salivary microbiota. However, Han et al. [2] argue that this conclusion is limited by a small sample size and two methodological inconsistencies, which could have influenced this result.

By reflecting the above cited positive as well as negative considerations with respect to premastication (summarised in Table 1), the interplay between the oral cavity (as a gateway/first point of contact with immunologically relevant sites) and the gut seems decisive but knowledge so far is limited. Here, the (maturation of the) oral‐gut axis seems to have a fundamental role concerning the initial bacterial colonization and its subsequent connection in health and disease. Especially, when it comes to the discussion of disease transmission versus beneficial effects on the host by stimulating the developing immune system of the infant, a better understanding of this interaction will also support deciphering the aetiology of atopic diseases. In parallel, the fading maternal passive immunity (excluding the possible Ig transfer via saliva or breastmilk) and the successive programming of the child's immune response to foreign stimuli leave a period of high modulation potential in which premastication of infant food takes place.

Studies investigating premastication have thus far consisted largely of case reports and comparative studies (see Figure 2 and Table S1) mostly driven by the “danger signal of pathogenic disease transmission”. Data gained from these studies have to be considered rather low in the hierarchy of evidence, therefore limiting their validity especially when it comes to uncovering mechanistic aspects or even confirming hypotheses. Evidently, premastication per se cannot be used in a blinded randomised controlled trial (RCT), resulting in a lack of reliability and validity compared to other RCTs.

## 7 Conclusions

To quote Pelto et al. [1] “if premastication poses significant dangers from the perspective of disease transmission, it is unlikely that it would have sustained through the course of cultural evolution.” Premastication is an underreported and under‐researched practice of feeding infants that is practiced across cultures since thousands of years as well as today. In the commentary on Pelto et al. [1], Lack and Penagos highlight the inverse relationship between the decline in the practice of premastication world‐wide and the “emerging epidemic of allergies and autoimmune diseases” (Back JF, 2002; Grundy J. 2002, as cited in the collection of discussions Van Esterik et al.) [15]. The small body of research that is available has largely focused on the risks of premastication, such as the transfer of infectious diseases, including HIV and HHV‐8, as well as being implicated in the transfer of bacteria known to cause early childhood caries and stomach ulcers. Whilst these concerns are valid, evidence suggests that overall, the risk of oral transfer of infectious microbes remains low [8, 89]. Additionally, measures such as the adult not pre‐chewing when having an open lesion in the mouth or when being sick can further reduce this risk. What has been neglected in recent years in our view is the focus on the benefits this feeding technique can provide: Passing on an “immune‐stimulating cocktail” of pre‐digested, insalivated food may support a gentler introduction of solids into the infant's diet (Figure 1). Further, this repeated transfer of the soft food portion (with an assumed very low risk of aspiration and choking), incidentally serves current food allergy prevention recommendations for oral tolerance development [31, 32], possibly supporting the introduction of a more diverse diet in infancy and including also so called “high‐allergenic” foods like peanuts or tree nuts. Increasing food diversity (implying high‐fibre foods) during infancy in turn is regarded as a potential method for allergy prevention [90, 91]. Moreover, for example for peanuts, it is (even) recommended to adapt this introduction in accordance to “normal” (usual family‐ /country‐typical) eating habits [31, 32].

This intensive microbial exposure from (mostly) mother to child that takes place during premastication could influence developmental imprinting of the child's immune system. With the growing evidence of the importance of the microbiome in the development of diseases [13, 73], and in light of the “evolved” hygiene hypothesis, premastication could support the development of a non‐delayed gut microbiome maturation [35] and diversification in infants (Figures 3 and 4). Premastication, if done in a safe way, could be a long‐practiced and well‐rehearsed method of counteracting the increasing incidence of atopic diseases in the Western world. In particular, it could support the development of food compatibility and even tolerance to specific food antigens, which in turn may prevent (food) allergies in children [44]. The reasons described above suggest that this traditional feeding practice should be investigated in greater depth.

## Author Contributions

Anne Steinberg, Sophie Goebel and Birgit Ahrens were involved in the conception and design of the review with further contributions by Tara Eckert, Lara Meixner, Katharina Blumchen, Meral Sturmfels and Kirsten Beyer. The manuscript was written by Anne Steinberg, Sophie Goebel, Tara Eckert and Birgit Ahrens. Stefan Schülke helped in creating Figures 1, 3 and 4. Submission to publication was finalised in close collaboration with all authors.

## Disclosure

Disclaimer: B. Ahrens and S. Schülke: The views expressed in this work are their personal views and may not be understood or quoted as being made on behalf of or reflecting the position of the respective national competent authority, the European Medicines Agency, or one of its committees or working parties.

## Conflicts of Interest

B. Ahrens receives research funding from the Federal Ministry of Food and Agriculture (BMEL); she reports participation in RCTs dealing with (hydrolyzed) infant formulas for allergy prevention (HiPP), the latter outside the submitted work. K. Beyer reports advisory board/consulting fees or speaker's bureau from Aimmune Therapeutics, Bencard, Danone/Nutricia, DBV, Hycor, Infectopharm, Mabylon, Meda Pharma/Mylan, Nestle, Novartis and ThermoFisher; and research grants from Aimmune, ALK, Danone/Nutricia, DBV Technologies, Hipp, Infectopharm and Novartis outside the submitted work. K. Blumchen reports advisory board/consulting fees or speaker's bureau from Aimmune Therapeutics, DBV Technologies, Novartis, Bencard Allergie, Stallergenes Greer, Thermofisher Scientific, Danone, Allergopharma, Mylan, Sanofi, Engelhard, ALK, Siemens Healthineers; and research grants from Novartis Pharma GmbH, Aimmune Therapeutics, DBV Technologies and Allergy Therapeutics outside the submitted work.

## Supporting information


**Table S1:** Listing of all publications appearing on a PubMed search in JUN 2024 using the terms “premastication OR pre‐mastication OR prechewing OR pre‐chewing”. All 68 results were generated, dated between 1948 and 2024. References are listed below, including the 12 papers not directly related to the feeding technique.

**Table S2:** Major findings of publications that are relevant for the discussion of the potential impact of premastication on allergy and microbiome development, sorted by topic (premastication, allergy and hygiene, microbiome and allergy and microbiome) and year.

## Data Availability

To be decided.
