# Supplementary material for: Premastication—Review of an Infant Feeding Practice and Its Potential Impact on Allergy and Microbiome Development
Source: Allergy. 2025 Sep 8;80(10):2726–37. doi: 10.1111/all.16676 (PMC12486369; doi:10.1111/all.16676)
Supplement: Supplementary file 1 — Table S1: Listing of all publications appearing on a PubMed search in JUN 2024 using the terms “premastication OR pre‐mastication OR prechewing OR pre‐chewing”. All 68 results were generated, dated between 1948 and 2024. References are listed below, including the 12 papers not directly related to the feeding technique. [file ALL-80-2726-s002.pdf]

Supplementary Table 1. Listing of all publications appearing on a PubMed search in JUN 2024 using the terms "premastication OR pre-mastication OR prechewing OR pre-chewing". In all 68 results were generated, dated between 1948 and 2024. References are listed below, including the 12 papers not directly related to the feeding technique.*

Not applicable (na) was chosen for those publications, which were not rated as relevant (column "Relevant yes/no"); see column "Summary/Abstract/Relevant Information about Premastication"; "View on Premastication", and "Study Type".

Year	Results by Year	Study	Topic	Summary findings/Mentions of Premastication	Relevant* (yes/ no)	View on Premastication* (positive/ negative/ indifferent/ mixture)	Article/ Study Type*
2024	1	Ismael-Mohammed et al.[1]	Mastication or Swallowing Impairments	na	no	na	na
2023	1	Ge et al.[2]	Caries	Irregular tooth brushing and always prechewing children's food were risk factors for ECC (P < 0.05).	yes	negative	Retrospective cohort study
2022	1	Nash et al.[3]	HIV	Case report of a 13-month-old Alaska Native child from rural Alaska who was diagnosed with HIV although the mother did not have HIV... 'A grandparent who had been acting as primary caregiver was discovered to be HIV-infected with detectable viral load resulting from intermittent nonadherence to her medication regimen. This grandparent reported feeding the child premasticated food'.	yes	negative	Case report
	2	Czarnik et al.[4]	Prevalence	Approximately 11.6% to 17.7% of caregivers reported prechewing as a food preparation practice between 7 and 13 months.	yes	indifferent	Retrospective multicenter study
2021	1	Ren et al.[5]	Feeding difficulty	Feeding difficulty occurred more often among children with picky eating behavior or whose caregivers once used the strategy of pre-mastication.	yes	negative	Cross-sectional multicenter study
	2	Yang et al.[6]	Syphilis	Feeding patterns and intimate contact in daily life may represent the main mode of nonsexual transmission of syphilis in children. The authors recommend that caregivers should be to discourage prechewing of food.	yes	negative	Review of case reports
2020	1	Susilorini et al.[7]	na	'Both mild and strong tahneeq increased the systemic CD8+ T-lymphocytes in neonatal Wistar rats. Both mild and strong tahneeq methods probably lead to neonatal immune tolerance by transferring the adult's microbiome into the neonatal gut to generate commensal bacteria colonization.'	(yes) - Thaneeq not	positive	In vivo study (animal model)

					preche wing		
	2	Janei et al.[8]	Feeding habbits of termite	na	no	na	na
	3	Myburgh et al.[9]	HIV	1 case reported premastication in total cohort of 32. Infection prevention breaches, re-use of medical devices, premastication and surrogate breastfeeding are likely to be major risk factors for horizontal HIV transmission in resource-limited settings.	yes	negative	Review of case reports
	4	Schaal et al.[10]	child development	‘There is circumstantial evidence that human infants and young children want to taste foods following carers’ oral food odours. Such mother-induced odour learning may be secondary to intentionally giving infants premasticated foods […]which exposes infants to pretreated highly odorous foodstuffs], whose novelty may be attenuated by the carer’s added saliva and other oral odour substrates (labial sebaceous glands, breath)’. However, it is not known whether human maternal saliva channels chemosensory information to offspring.	(yes) - only mentio n of prema sticatio n, not main theme	indifferent	Review
	5	Badescu et al.[11]	Evolution	Chimpanzee mothers practiced premastication with infants aged 4-6 years, and were more likely to do so for the younger offspring. The mothers also were more likely to practice premastication if they were not first time mothers. Both easy to chew and tougher foods were shared. Premastication may be an infant-rearing strategy that facilitates the transition from a diet of exclusive maternal milk to solid food during early infancy. It ‘may provide energetic, immune, or growth benefits to infants through reduced chewing effort and maternal saliva.’	yes	positive	Animal observational study
2019	1	Babatola et al.[12]	H. pylori	‘H. pylori infection seroprevalence rate was 32.8% and increased with age. Living in one room accommodation, large families, playing with soil, family history of dyspepsia, practice of premastication, sharing of plates and cutlery, and water closet toilet were associated with H. pylori Ig G seropositivity ($p<0.05$) on binary regression analysis. 50 of 68 of H. pylori seropositive chidlren practiced premastication, compared to 75 of 167 H. pylori seronegative.’	yes	negative	Cross-sectional study / Seroepidemiol ogical survey
	2	Kittisakmon tri et al.[13]	Prevalence	‘Unfavourable behaviours such as premastication (pre-chewing food) and using food as a reward were also more frequent in older children’, ‘Although there are theoretical benefits of premastication, pending availability of more robust data families should be advised to avoid it, especially in countries where HIV infection is prevalent.'	yes	negative	Cross-sectional study
2018	1	Okanda et al.[14]	HIV	Premastication was less prevalent among mothers with HIV (3.9% vs. 13.2%, p = 0.001) who were also more knowledgeable about potential risk of HIV transmission through premastication (83.1% vs 71.2% p = 0.005).	yes	indifferent	Cross-sectional study

	2	Xiong et al.[15]	Areca nuts	na	no	na	na
	3	Ho et al.[16]	Dental cements	na	no	na	na
	4	Conkle et al.[17]	Prevalence, risks and benefits	Premastication is common in Laos. Premastication was most common for children 6–13 months of age, and up to 2 years of age. Across all age groups, infants and children that received premasticated food had lower LAZ than those that did not receive premasticated food 'if premastication affects infant health and nutrition, it is affecting a large percentage of infants in Laos'	yes	negative/indifferent	Cross-sectional study
	5	Zhao et al.[18]	Prevalence, risks and benefits	The overall prevalence of premastication was 26.9% and varies from 14–43% among the 8 cities. Premastication was not associated with occurrences of illness or with the nutritional indicators of height-for-age Z score, weight-for-age Z score, weight-for-height Z score, head circumference Z score and hemoglobin (P all >.05). Premastication occurred more often among infants whose mothers' education was lower (P < .001),	yes	positive/indifferent	Multicentre cross-sectional study
2017	1	Crabtree et al. [19]	Prevalence	3 of 270 (1.1%) premasticated food, however the study showed that sharing utensils can lead to HHV-8 transmission (Independent risk factors for HHV-8 incident infection included having a child who shared utensils with a primary caregiver. Sharing utensils was observed in 10 of 270 cases.	yes	indifferent	Prospective cohort study
2016	1	Martin et al.[20]	Prevalence	In initial interviews, 84% and 51% of non-exclusively breastfed mothers (n = 132), respectively, reported having ever premasticated solids or orally transferring liquids. Premastication and orally transferring liquids were reported in 54% and 9%, respectively. Early CF risks may be minimized by premastication and continued intensive breastfeeding	yes	positive	Cross-sectional study
	2	Han et al.[21]	Impact on microbiome	Salivary microbiota of Tsimane infants and young children up to two years of age do not appear closely related to those of their mothers, despite frequent premastication and preliminary evidence that maternal bacteria is transmitted to premasticated foods.	yes	positive/indifferent	Comparative study
	3	Zhang and Jin[22]	Syphilis	Case study of infant who had acquired early syphilis which had been transmitted by prechewed food from his grandmother. 'Prechewing food is a custom in most parts of China. Prechewing an infant's food could be an avenue of disease transmission, although this is not fully recognized.'	yes	negative	Case report
	4	Habicht and Pelto[23]	Risks and benefits	'The research community concerned with premastication has only started on a productive research route. We do not yet have plausible stories of causality against or in favour of premastication, and in what population groups.'	yes	indifferent	Comment

	5	Conkle et al.[24]	Diarrhoea disease	After adjusting for covariates, including breastfeeding and consuming sweets and dairy, prechewing was associated with a 58% higher risk of 2-week diarrhoea prevalence. Prechewing of infant food is associated with increased diarrhoea among 10-month-old infants.	yes	negative	Retrospective cross-sectional study
	6	Finnegan et al.[25]	ECC	'Specific detrimental behaviours reported by the participants included pre-chewing food for their child and providing their children with bottles containing cariogenic liquids in their cribs or beds.'	yes	negative	Multicentre comparative study
	7	Nyati-Jokomo et al.[26]	HIV	'Emerging themes relating to risky practices were rituals surrounding […] premastication. These practices exposed babies to bodily fluids such as saliva, […] which in turn put the babies at low to high risk of contracting HIV.'	yes	negative	Cross-sectional study
2015	1	Ding et al.[27]	H. pylori	'Consuming meals in unsanitised conditions, sharing towels, receiving pre-chewed food from the mother, artificial feeding and family history of gastrointestinal disease were significantly associated with the risk of infection.'	yes	negative	Prospective, cross-sectional, multicenter study
2014	1	Auer-Hackenberg et al.[28]	Prevalence	A quarter (n = 20, 24%) of 82 participants stated to perform premastication regularly. Premastication is a common practice in these Central African communities. 'Since the risk–benefit balance has not been conclusively assessed, further prospective studies are needed to develop evidence-based recommendations for this feeding practice in sub-Saharan African communities.'	yes	indifferent	Cross-sectional study
2013	1	Dewey[29]	Benefits	'Premastication allows infants to consume the same foods as consumed by their mothers and transfers various constituents of saliva (similar to those in breast milk) that may have healthpromoting effects. If one assumes that, in addition to breast milk, infants of hunter-gatherers received premasticated complementary foods in the same proportions as consumed by their mothers, it is possible to construct a hypothetical nutrient profile of this composite diet.'	yes	positive/indifferent	Opinion/Review
	2	Nesheim et al.[30]	HIV	'There are the so-called "late" perinatal transmissions, which may result from infants ingesting "premasticated" food, from being breastfed by an HIV-infected woman.'	yes	negative	Review
	3	Pattanaporn et al.[31]	ECC	Children's age, MS colonization, and mothers' prechewing feeding habits were the most significant risk indicators for ECC in Thai children. Mothers who gave birth vaginally were more likely to prechew food to feed their children than those mothers who gave birth by C-section (3-year-old group, P = 0.015).	yes	negative	Comparative study
	4	Labraña et al.[32]	HIV	Case report which suggests that HIV can be transmitted through the consumption of foods that have been premasticated by a person infected with HIV. 'Premastication is a transmission path that had not been reported,	yes	negative	Case report

				being a possible explanation for some cases of late transmission of HIV in infants, so far attributed to breastfeeding.’			
	5	Gaur et al.[33]	HIV	The practices of prechewing/pre- warming of food for HIV-exposed children persist at a low rate, despite informing about the risk multiple times. ‘With no clear benefit of these practices and the potential for transmission of HIV and other infections, care providers should be counseled against prechewing/prewarming food for their children.’	yes	negative	Letter/ Prospective cohort multicenter study Letter
2012	1	Cotton et al.[34]	HIV	‘Factors such as poor injection safety, undocumented surrogate breast feeding, an HIV-infected adult feeding premasticated food to a weaning toddler, poor hygienic practice in the home and using unsterilised equipment for minor surgical or traditional procedures are of cumulative concern.’	yes	negative	Comment
	2	Ivy 3rd et al.[35]	HIV, prevalence	Comparison of 11 case-patients and 35 HIV-exposed controls of similar age. Sixteen (35%) of 46 children were fed premasticated food, 10 (22%) by an HIV-infected caregiver. Twenty-seven percent of case-patients received premasticated food from an HIV-infected caregiver compared with 20% of controls. In the cross-sectional investigation, 48 (31%) of 154 primary caregivers of children aged ≥6 months reported the children received premasticated food from themselves or someone else. The prevalence of premastication decreased with increasing caregiver age and had been used to feed children aged 1-36 months.	yes	negative	Case-control and cross-sectional multicenter study
2011	1	Maritz et al.[36]	HIV, prevalence	‘There were 106 (69%) caregivers who practiced premastication. The median age of infants who received premasticated food was 6 (interquartile range: 4–6) months. Forty-six (43%) infants were teething, and 44 (42%) had oral mucosal lesions while receiving premasticated food. Fifty-five (52%) caregivers reported an oral condition, mostly bleeding gums, mouth sores, and thrush, and 41 (39%) caregivers reported blood in the food. Premasticating caregivers had a significantly lower educational level than those caregivers who did not engage in this practice. Premastication practices were cultural (40%), habit (20%), and on mother's advice (75%). Reasons for premastication were to pretaste (68%), encourage eating (61%), estimate food temperature (85%), and homogenize food (60%).. […] Education should include advice to avoid premastication and to seek health advice for oral conditions in the caregiver and child.’	yes	negative	Cross-sectional study
	2	Gaur et al.[37]	HIV, prevalence	‘Prechewing food, a potential risk factor for HIV transmission, and orally prewarming food, which has not been associated with HIV transmission but might expose a child to blood from an HIV- infected adult, are not uncommon practices in Latin America.’	yes	negative	Cross-sectional multicenter study
	3	Centers for Disease	HIV, prevalence	Most caregivers were HIV-infected, ‘posing a potential risk for HIV transmission to children in their care who are uninfected. Furthermore, the	yes	negative	Cross-sectional study

		Control and Prevention [38]		higher prevalence of premastication among black and younger caregivers suggests the need for targeted prevention messages for these populations.'			
	4	Hafeez et al.[39]	HIV, prevalence	Practicing premastication is a common practice among HIV-infected mothers in the Bronx. Mothers with childhood exposure to premastication were about 7 times more likely to ever have used this feeding practice compared with mothers who denied having received prechewed food as a child. 'health care providers involved in the health care of families affected by HIV should consider asking questions regarding premastication when discussing complementary feeding practices for infants and should offer advice about its advantages and possible risks.'	yes	negative/indifferent	Cross-sectional study
	5	Levison et al.[40]	Risks	no abstract available	yes	negative	Comment
2010	1	Dinubile[41]	HIV	'Compared with exclusive breastfeeding, it seems biologically plausible that premastication may be an incremental HIV risk factor for a substantial number of infants given mixed feedings[…] On the other hand, pending more quantitative controlled data, counseling nursing mothers against prechewing their babies' food during weaning might potentially cause more harm than good.'	yes	indifferent	Correspondence
	2	Castilho and Barros Filho[42]	History/prevalence	'During the preindustrial period, customs varied little and the likelihood of survival was linked to breastfeeding or its substitution by a wetnurse's milk. Where this was not possible, infants were given animal milk, pre-chewed foods or paps that were poor in nutrients and contaminated, which caused high mortality rates.'	yes	indifferent	Review
	3	Frange et al.[43]	HIV	No known mode of HIV transmission (including breastfeeding or use of premasticated food) could be found.	yes	indifferent	Multicenter study
	4	Butler et al.[44]	Prevalence/ pathogens	'African children are exposed to saliva through a variety of acts, practised by a variety of caregivers, with no single predominant practice. This diversity poses challenges for epidemiologic work seeking to identify specific saliva-passing practices that transmit viruses.'	yes	negative	Population-based survey
	5	Pelto et al.[45]	Prevalence, risks and benefits	'We conclude with the argument for a concerted research effort to determine whether premastication can solve not only the 'weanling dilemma' in poor countries but also some of the health problems among the better-off.'	yes	positive	Article
	6	Van Esterik et al.[46]	Risks and benefits	Note: Abstract not applicable, this is a collection of 5 contextualising commentaries on the paper Pelto et al. 2010	yes	mixture	Comments
	7	Aggett[47]	Risks and benefits	'It is probably not likely that we will come to a point of encouraging mothers to practise premastication, but the topic does open up opportunities to explore what seems to have been and to still be a common practice and to	yes	indifferent	Comment

				reflect on the lessons that might be brought to bear on current policy and advice on feeding in infancy and early childhood.'			
2009	1	Gaur et al.[48]	HIV	Case study of 3 children who were fed food on multiple occasions that had been premasticated by a care provider infected with HIV; in 2 cases concurrent oral bleeding in the premasticating adult was described. 'Phylogenetic analyses supported the epidemiologic conclusion that the children were infected through exposure to premasticated food from a caregiver infected with HIV in 2 of the 3 cases.'	yes	negative	Case Reports
	2	Zhou et al.[49]	Syphilis	Case report of 2 cases of infantile syphilis transmitted by mouth-to-mouth feeding from actively infected relatives.	yes	negative	Case Reports
2008	1	Qin et al.[50]	ECC	Forty-six S-ECC children but only 2 caries-free children received prechewed food ($P < .001$)	yes	negative	Comparative study
2007	1	Harrison et al.[51]	ECC	Exploratory analyses revealed that rates of dmfs were higher in children whose mothers had: (1) prechewed their food; (2) been raised in a rural environment; and (3) a higher family income ($P<.05$).	yes	negative	Randomized controlled trial
2006	1	van Palenstein Helderman et al.[52]	ECC	A retrospective cohort study on ECC and associated factors was conducted among mothers with 25- to 30-month-old infants in a community where prolonged breastfeeding was common practice. All infants who consumed sugary supplementary food or rice that was pre-chewed by the mother, or who fell asleep with the breast nipple in their mouths, had ECC.	yes	negative	Retrospective cohort study
2005	1	Mohri et al.[53]	Gum chewing and neurosience	na	no	na	na
2004	1	Bloemena[54]	EBV	no abstract available	nd	na	na
2003	1	Mégraud[55]	H. pylori	'In developing countries the fecal-oral route is plausible, in addition to the oral-oral route; indeed, diarrhea is common, fecal hygiene is rarely performed and water is not treated. Furthermore, when vomiting occurs, hygienic practice cannot be adequate due to the unsatisfactory sanitary conditions at home, and certain habits, e.g. premastication of food, are frequent.'	yes	negative/in different	Review
1999	1	Qureshi et al.[56]	H. pylori	H. pylori exposure rate increased with the advancement of age and lowering of socioeconomic status. Early exposure might be related to the use of premasticated food by the mothers for feeding of children; dental plaque being the reservoir of infection in adults.	yes	negative/in different	Comparative study
1998	1	Yagyu et al.[57]	Gum chewing and neurosience	na	no	na	na
1997	1	Yagyu et al.[58]	Gum chewing and neurosience	na	no	na	na

1995	1	Imong et al.[59]	Bacterial contamination	Bottle feeding, premastication, and mashing were significantly related to an increased bacterial content of weaning foods in 62 mother-infant pairs in Chiang Mai, while boiling foods to make soups, preparing in and feeding from a banana leaf, and using boiled water to prepare foods all reduced their bacterial content.	yes	negative	Longitudinal study
1993	1	Walburn and Pergam[60]	Prevalence	'Two recent JOURNAL articles mentioned the relatively common parenting practices of prechewing food for infants and parental nose blowing. We had previously reported a number of such child care practices among American black families.'	yes	indifferent	Comment
1992	1	Steinkuller et al.[61]	Streptococcal pharyngitis	Three infants with group A streptococcal pharyngitis were found to have eaten food prechewed by their parents. One parent had a history of recent pharyngitis and another had frequent episodes of tonsillitis. Prechewing of babies' food, which may be more common in the United States than is generally recognized, may be a mode of transmission of group A streptococcal pharyngitis to infants.	yes	negative	Case Reports
1990	1	Huang[62]	HBV	Premastication seemed to be a possible important risk factor in the family transmission of HBV in a sample of 520 preschool children aged 2-6 in Shanghai, China.	yes	negative	Seroepidemiol ogical survey
1989	1	Lynn[63]	Orthodontics	na	no	na	na
	2	Neander and Morse[64]	Prevalence	Comparison of traditional and present-day infant feeding practices of the Northern Alberta Woodland Cree.<br>Both the traditional and present-day mothers introduced solid foods relatively early, but the traditional mothers premasticated the infants' food rather than using commercial baby food or a blender.	yes	indifferent	Comparative study
	3	Imong et al.[65]	Bacterial contamination	Samples of weaning food and supplementary water were collected for analysis of bacterial content from among a random sample of 65 infants under the age of 1 year living in a rural area of northern Thailand. Weaning foods were contaminated during preparation, in part through premastication and also via mode of cleaning of utensils.	yes	negative	Random sampling study
1985	1	Tappen[66]	Paleopathology	na	no	na	na
1981	1	Svare et al.[67]	Dental cements	na	no	na	na
1948	1	Peiper[68]	not known	no abstract available	nd	na	na

Abbreviations: Human immunodeficiency virus (HIV); Early Childhood Caries (ECC); Helicobacter pylori (H. pylori); not applicable (na); non-determinable (nd)

Notes:

The publication by Czarnik et al.4 is listed in PuBMed, Presentation "Results by Year", in 2021 and 2022. Here, this publication is only listed in 2022.

*Disclaimer: The information presented in these columns is not guaranteed to be complete or accurate, and reflects solely the personal opinions of the authors

References:

1. Ismael-Mohammed K, Bolivar-Prados M, Laguna L, Nunez Lara A, Clave P. Exploring Texture and Biomechanics of Food Oral Processing in Fork-Mashable Dishes for Patients with Mastication or Swallowing Impairments. *Foods*. 2024;13(12)
2. Ge X, Lyu X, Zhou Z, et al. Caesarean-section delivery and caries risk of 3-year-old Chinese children: a retrospective cohort study. *BMC Oral Health*. 2023;23(1):373.
3. Nash SH, Rutledge M, Frenkel LM, et al. HIV Transmission Through Premastication. *Pediatrics*. 2022;150(4)
4. Czarnik M, Hamner HC, Moore LV. Food Preparation Practices for Infants Aged From 7 to 13 Months. *J Nutr Educ Behav*. 2022;54(1):28-35.
5. Ren Z, Lan H, Szeto IM, et al. Feeding Difficulty Among Chinese Toddlers Aged 1-3 Years and Its Association With Health and Development. *Front Pediatr*. 2021;9:758176.
6. Yang WJ, Hu HH, Yang Y, Li JH, Guo H. Unusual erythematous plaque with white scales, a case of acquired syphilis in a child and literature review. *BMC Infect Dis*. 2021;21(1):528.
7. Susilorini, Suradi, Indarto D, Wasita B, Palupi PD. Immunomodulation of tahneeq method in IL-12 and CD8+ T-Lymphocyte, an in-vivo study in neonatal rats. *Saudi J Biol Sci*. 2020;27(10):2645-2650.
8. Janei V, Haifig I, Schonhaus GC, Costa-Leonardo AM. Gut Content and Laboratory Survival of the Termite Cornitermes cumulans (Isoptera: Termitidae: Syntermitinae) with Different Diets Including Nest Stored Food. *Neotrop Entomol*. 2020;49(5):677-684.
9. Myburgh D, Rabie H, Slogrove AL, Edson C, Cotton MF, Dramowski A. Horizontal HIV transmission to children of HIV-uninfected mothers: A case series and review of the global literature. *Int J Infect Dis*. 2020;98:315-320.
10. Schaal B, Saxton TK, Loos H, Soussignan R, Durand K. Olfaction scaffolds the developing human from neonate to adolescent and beyond. *Philos Trans R Soc Lond B Biol Sci*. 2020;375(1800):20190261.
11. Bădescu I, Sicotte P, Sandel AA, et al. Premasticated food transfer by wild chimpanzee mothers with their infants: Effects of maternal parity, infant age and sex, and food properties. *J Hum Evol*. 2020;143:102794.
12. Babatola AO, Akinbami FO, Adeodu OO, Ojo TO, Efere MO, Olatunya OS. Seroprevalence and determinants of Helicobacter pylori infection among asymptomatic under-five children at a Tertiary Hospital in the South-Western region of Nigeria. *Afr Health Sci*. 2019;19(2):2082-2090.
13. Kittisakmontri K, Fewtrell M, Roekworachai K, Phanpong C, Lanigan J. Complementary feeding: Attitudes, knowledge and practices of urban families in northern Thailand. *Nutr Diet*. 2019;76(1):57-66.
14. Okanda J, Otieno G, Kinuthia J, Kohler P, John-Stewart G. Higher likelihood of 6-months exclusive breastfeeding among HIV infected than uninfected mothers: a household survey in Kenya. *Int Breastfeed J*. 2018;13:51.
15. Xiong X, Hou A, Yi S, et al. Analysis of oral microorganism diversity in healthy individuals before and after chewing areca nuts using PCR-denatured gradient gel electrophoresis. *Anim Nutr*. 2018;4(3):294-299.
16. Ho TK, Satterthwaite JD, Silikas N. The effect of chewing simulation on surface roughness of resin composite when opposed by zirconia ceramic and lithium disilicate ceramic. *Dent Mater*. 2018;34(2):e15-e24.
17. Conkle J, Kounnavong S, Young M, Stein AD. Premastication and length for age among children under 24 months in Laos. *Matern Child Nutr*. 2018;14(1)
18. Zhao A, Zheng W, Xue Y, et al. Prevalence of premastication among children aged 6-36 months and its association with health: A cross-sectional study in eight cities of China. *Matern Child Nutr*. 2018;14(1)
19. Crabtree KL, Wojcicki JM, Minhas V, Kankasa C, Mitchell C, Wood C. Association of Household Food- and Drink-Sharing Practices With Human Herpesvirus 8 Seroconversion in a Cohort of Zambian Children. *J Infect Dis*. 2017;216(7):842-849.

20. Martin MA, Garcia G, Kaplan HS, Gurven MD. Conflict or congruence? Maternal and infant-centric factors associated with shorter exclusive breastfeeding durations among the Tsimane. *Soc Sci Med*. 2016;170:9-17.
21. Han CS, Martin MA, Dichosa AEK, et al. Salivary microbiomes of indigenous Tsimane mothers and infants are distinct despite frequent premastication. *PeerJ*. 2016;4:e2660.
22. Zhang RZ, Jin HL. Syphilis in an Infant Acquired by Mouth-to-Mouth Transfer of Prechewed Food. *Pediatr Dermatol*. 2016;33(6):e344-e345.
23. Habicht JP, Pelto GH. Addressing epidemiological and public health analytic challenges in outcome and impact research: a commentary on 'Prechewing Infant Food, Consumption of Sweets and Dairy and Not Breastfeeding are Associated with Increased Diarrhea Risk of Ten Month Old Infants'. *Matern Child Nutr*. 2016;12(3):625-31.
24. Conkle J, Ramakrishnan U, Freeman MC. Prechewing infant food, consumption of sweets and dairy and not breastfeeding are associated with increased diarrhoea risk of 10-month-old infants in the United States. *Matern Child Nutr*. 2016;12(3):614-24.
25. Finnegan DA, Rainchuso L, Jenkins S, Kierce E, Rothman A. Immigrant Caregivers of Young Children: Oral Health Beliefs, Attitudes, and Early Childhood Caries Knowledge. *J Community Health*. 2016;41(2):250-7.
26. Nyati-Jokomo Z, January J, Ruparanganda W, Chitsike I. Risky traditional practices and prevention of mother-to-child transmission of HIV: the case of Chiota community in Zimbabwe. *AIDS Care*. 2016;28(1):52-6.
27. Ding Z, Zhao S, Gong S, et al. Prevalence and risk factors of Helicobacter pylori infection in asymptomatic Chinese children: a prospective, cross-sectional, population-based study. *Aliment Pharmacol Ther*. 2015;42(8):1019-26.
28. Auer-Hackenberg L, Thol F, Akerey-Diop D, et al. Short report: premastication in rural Gabon--a cross-sectional survey. *J Trop Pediatr*. 2014;60(2):154-6.
29. Dewey KG. The challenge of meeting nutrient needs of infants and young children during the period of complementary feeding: an evolutionary perspective. *J Nutr*. 2013;143(12):2050-4.
30. Nesheim S, Harris LF, Lampe M. Elimination of perinatal HIV infection in the USA and other high-income countries: achievements and challenges. *Curr Opin HIV AIDS*. 2013;8(5):447-56.
31. Pattanaporn K, Saraithong P, Khongkhunthian S, et al. Mode of delivery, mutans streptococci colonization, and early childhood caries in three- to five-year-old Thai children. *Community Dent Oral Epidemiol*. 2013;41(3):212-23.
32. Labraña Y, Alvarez AM, Villarroel J, Wu E. [Premastication: a new way of transmitting HIV. First pediatric case reported in Chile]. *Rev Chilena Infectol*. 2013;30(2):221-2. Premasticación: una nueva forma de transmisión del virus de inmunodeficiencia humana. Primer caso pediátrico informado en Chile.
33. Gaur AH, Cohen RA, Read JS, et al. Prechewing and prewarming food for HIV-exposed children: a prospective cohort experience from Latin America. *AIDS Patient Care STDS*. 2013;27(3):142-5.
34. Cotton MF, Marais BJ, Andersson MI, et al. Minimizing the risk of non-vertical, non-sexual HIV infection in children--beyond mother to child transmission. *J Int AIDS Soc*. 2012;15(2):17377.
35. Ivy W, 3rd, Dominguez KL, Rakhmanina NY, et al. Premastication as a route of pediatric HIV transmission: case-control and cross-sectional investigations. *J Acquir Immune Defic Syndr*. 2012;59(2):207-12.
36. Maritz ER, Kidd M, Cotton MF. Premasticating food for weaning African infants: a possible vehicle for transmission of HIV. *Pediatrics*. 2011;128(3):e579-90.
37. Gaur AH, Freimanis-Hance L, Dominguez K, et al. Knowledge and practice of prechewing/prewarming food by HIV-infected women. *Pediatrics*. 2011;127(5):e1206-11.
38. Centers for Disease Control and Prevention (CDC). Premastication of food by caregivers of HIV-exposed children--nine U.S. sites, 2009-2010. *MMWR Morb Mortal Wkly Rep*. 2011;60(9):273-5.
39. Hafeez S, Salami O, Alvarado M, Maldonado M, Purswani M, Hagmann S. Infant feeding practice of premastication: an anonymous survey among human immunodeficiency virus-infected mothers. *Arch Pediatr Adolesc Med*. 2011;165(1):92-3.
40. Levison J, Gillespie SL, Montgomery E. Think twice before recommending pre-masticated food as a source of infant nutrition. *Matern Child Nutr*. 2011;7(1):104; author reply 105-6.
41. Dinubile MJ. Premastication: a possible missing link? *Clin Infect Dis*. 2010;51(2):252-3.
42. Castilho SD, Barros Filho AA. The history of infant nutrition. *J Pediatr (Rio J)*. 2010;86(3):179-88.
43. Frange P, Burgard M, Lachassinne E, et al. Late postnatal HIV infection in children born to HIV-1-infected mothers in a high-income country. *Aids*. 2010;24(11):1771-6.
44. Butler LM, Neilands TB, Mosam A, Mzolo S, Martin JN. A population-based study of how children are exposed to saliva in KwaZulu-Natal Province, South Africa: implications for the spread of saliva-borne pathogens to children. *Trop Med Int Health*. 2010;15(4):442-53.
45. Pelto GH, Zhang Y, Habicht JP. Premastication: the second arm of infant and young child feeding for health and survival? *Matern Child Nutr*. 2010;6(1):4-18.

46. Van Esterik P, Williams A, Fewtrell MS, Tolboom JJ, Lack G, Penagos M. Commentaries on Premastication: the second arm of infant and young child feeding for health and survival? By Gretel Pelto, Yuanyuan Zhang & Jean-Pierre Habicht. *Matern Child Nutr*. 2010;6(1):19-26.
47. Aggett P. Premastication. *Matern Child Nutr*. 2010;6(1):2-3.
48. Gaur AH, Dominguez KL, Kalish ML, et al. Practice of feeding premasticated food to infants: a potential risk factor for HIV transmission. *Pediatrics*. 2009;124(2):658-66.
49. Zhou P, Qian Y, Lu H, Guan Z. Nonvenereal transmission of syphilis in infancy by mouth-to-mouth transfer of prechewed food. *Sex Transm Dis*. 2009;36(4):216-7.
50. Qin M, Li J, Zhang S, Ma W. Risk factors for severe early childhood caries in children younger than 4 years old in Beijing, China. *Pediatr Dent*. 2008;30(2):122-8.
51. Harrison R, Benton T, Everson-Stewart S, Weinstein P. Effect of motivational interviewing on rates of early childhood caries: a randomized trial. *Pediatr Dent*. 2007;29(1):16-22.
52. van Palenstein Helderman WH, Soe W, van 't Hof MA. Risk factors of early childhood caries in a Southeast Asian population. *J Dent Res*. 2006;85(1):85-8.
53. Mohri Y, Fumoto M, Sato-Suzuki I, Umino M, Arita H. Prolonged rhythmic gum chewing suppresses nociceptive response via serotonergic descending inhibitory pathway in humans. *Pain*. 2005;118(1-2):35-42.
54. Bloemena E. [Pre-chewed...]. *Ned Tijdschr Tandheelkd*. 2004;111(8):316. Voorgekaud...
55. Mégraud F. [When and how does Helicobacter pylori infection occur?]. *Gastroenterol Clin Biol*. 2003;27(3 Pt 2):374-9. Quand et comment s'infecte-t-on par Helicobacter pylori?
56. Qureshi H, Hafiz S, Medhi I. H. pylori IgG antibodies in children. *J Pak Med Assoc*. 1999;49(6):143-4.
57. Yagyu T, Kondakor I, Kochi K, et al. Smell and taste of chewing gum affect frequency domain EEG source localizations. *Int J Neurosci*. 1998;93(3-4):205-16.
58. Yagyu T, Wackermann J, Kinoshita T, et al. Chewing-gum flavor affects measures of global complexity of multichannel EEG. *Neuropsychobiology*. 1997;35(1):46-50.
59. Imong SM, Jackson DA, Rungruengthanakit K, et al. Maternal behaviour and socio-economic influences on the bacterial content of infant weaning foods in rural northern Thailand. *J Trop Pediatr*. 1995;41(4):234-40.
60. Walburn J, Pergam J. Prechewing food and parental nose blowing. *J Pediatr*. 1993;122(5 Pt 1):835-6.
61. Steinkuller JS, Chan K, Rinehouse SE. Prechewing of food by adults and streptococcal pharyngitis in infants. *J Pediatr*. 1992;120(4 Pt 1):563-4.
62. Huang MJ. [An epidemiological study on prevalence and risk factors of hepatitis B virus (HBV) infection in preschool children]. *Zhonghua Liu Xing Bing Xue Za Zhi*. 1990;11(3):129-32.
63. Lynn JM. The Lynn Maxilla Rotator Combination Appliance and Lynn Archial Face Bow. *Funct Orthod*. 1989;6(4):4-13.
64. Neander WL, Morse JM. Tradition and change in the Northern Alberta Woodlands Cree: implications for infant feeding practices. *Can J Public Health*. 1989;80(3):190-4.
65. Imong SM, Rungruengthanakit K, Ruangyuttikarn C, Wongsawasdii L, Jackson DA, Drewett RF. The bacterial content of infant weaning foods and water in rural northern Thailand. *J Trop Pediatr*. 1989;35(1):14-8.
66. Tappen NC. The dentition of the "old man" of La Chapelle-aux-Saints and inferences concerning Neandertal behavior. *Am J Phys Anthropol*. 1985;67(1):43-50.
67. Svare CW, Peterson LC, Reinhardt JW, et al. The effect of dental amalgams on mercury levels in expired air. *J Dent Res*. 1981;60(9):1668-71.
68. Peiper A. [Pre-chewing]. *Arch Kinderheilkd*. 1948;135(2):67-70. Das Vorkauen.
