# Supplementary material for: Premastication—Review of an Infant Feeding Practice and Its Potential Impact on Allergy and Microbiome Development
Source: Allergy. 2025 Sep 8;80(10):2726–37. doi: 10.1111/all.16676 (PMC12486369; doi:10.1111/all.16676)
Supplement: Supplementary file 2 — Table S2: Major findings of publications that are relevant for the discussion of the potential impact of premastication on allergy and microbiome development, sorted by topic (premastication, allergy and hygiene, microbiome and allergy and microbiome) and year. [file ALL-80-2726-s001.pdf]

Supplementary Table 2. Major findings of publications that are relevant for the discussion of the potential impact of premastication on allergy and microbiome development, sorted by topic (Premastication, Allergy and Hygiene, Microbiome and Allergy and Microbiome) and year.*

Topic	Publication	Major findings
**Premastication: risks and benefits**	Pelto et al. (2010)[1] #, §	- Premastication is reported by one third of global ethnographies with data on infant feeding (n=119) in the electronic version of HRAF (Human Relations Area Files).<br>- 63% of Chinese university students (75 of 104) reported that they received premasticated food as infants, especially meat, rice, other grains, 'tough foods', and nuts.<br>- Potential benefits of premastication include the digestive (e.g. amylases) and immunological (antimicrobial, anti-inflammatory and growth factors) properties of saliva, increasing the nutrient supply, and strengthening the mother-child-bond.<br>- The transmission of pathogens via saliva is a potential risk of premastication.
	Van Esterik et al. (2010)[15], &	5 commentaries on the paper Pelto et al. 2010 that reflect different opinions about premastication:<br>- **Van Esterik** highlights the importance of interdisciplinary research (anthropology and nutrition) to address maternal and child nutrition.<br>- **Williams** emphasises the need of further research to clarify the effects of premastication.<br>- **Fewtrell** points to the potential of promoting immune tolerance to premasticated allergenic foods, but calls for an extensive risk-benefit analysis, particularly with regard to the risk of HIV infection.<br>- **Tolboom** confirms the occurence of premastication in certain cultures. However, the promotion of this practice in other cultures is not advantageous in his eyes.<br>- **Lack and Penagos** refer to the cytokines, chemokines, antibodies, and other molecules in saliva that could play a role in the development of oral tolerance to dietary antigens.
	Conkle et al. (2016)[3],‡	- Children (aged 10 months, n=203) who received prechewed food had a higher diarrhoea prevalence compared to those who did not (n=1567).<br>- Prechewing was associated with a 58% higher risk of 2 week diarrhoea prevalence, whereas continued breastfeeding at the age of 10 months showed a reduced risk for diarrhoea.
	Habicht and Pelto (2016)[4],&	Comment on Conkle et al. (2016):<br>- Recommendations against premastication based on limited data should be avoided.<br>- To actually discourage premastication to prevent diarrhoea requires a detailed consideration of other causes of diarrhoea and of the benefits of premastication.
**Premastication: impact on oral microbiome**	Han et al. (2016)[2], §	- Tsimane mothers practice premastication in the first two years of their infant's life. Especially with foods that are considered too hot, too dry, or that pose a choking hazard.

		- Salivary microbiotas of 12 related mother-infant pairs, which shared premasticated food, were no more similiar than those of unrelated pairs.
**Premastication: immunomudlation**	Susilorini et al. (2020)[57], ¶	- Traditional method of Tahneeq (rubbing the palatal mucosa of newborns with premasticaed Ajwa palm dates) increases CD8+ T-lymphocytes, and IL-12 expression in the palatal and gingival mucosa in neonates of Wistar rats. This might contribute to the stimulation and development of the immune system.
**Allergy and hygiene: saliva contact**	Hesselmar et al. (2013)[20], †	- Children from parents who cleaned their child's pacifier by sucking it (n=65) were less likely to have asthma, eczema, and sensitisation at 18 months of age than those who did not clean the pacifier this way (n=58). This effect remained for eczema at 36 months of age.<br>- Vaginal delivery also showed protective effects on eczema development.<br>- The salivary microbiota differed between children whose parents cleaned their pacifier by sucking it compared to those who did not use this cleaning technique, likely being involved in the immune stimulation and subsequent reduction of risk in these atopic diseases.
	Soriano et al. (2021)[79], †	- Using a pacifier at 6 months was associated with food allergy development at 12 months (n=894), but not pacifier use at other ages.<br>- Persistent and repeated antiseptic use over the first 6 months was associated with higher food allergy risk.<br>- Not cleaning the pacifier with antiseptic at 6 months was not associated with food allergy development.
	Kubo et al. (2023)[88], ‡	- 9.9 % (n=336) of 3,380 Japanese children were fed using shared eating utensils and 2.2 % (n=76) used a pacifier, which was cleaned by parental sucking.<br>- Children who shared eating utensils during infancy with their parents or whose parents sucked their pacifier for cleaning were significantly less likely to have eczema at an average age of 9-13 years.
**Allergy and hygiene: infectious microbes contact**	Matricardi et al. (2000)[77], ‡,§	- Lower prevalence of antibodies against orofecal and foodborne microbes (*Toxoplasma gondii*, *hepatitis A virus*, and *Helicobacter pylori*) in the serum of atopic patients (n=240) compared to controls (n=240).<br>- Participants exposed to at least two orofecal and foodborne infections (*H. pylori*, *T. gondii*, *Hep. A*) also had lower levels of allergic asthma and allergic rhinitis.<br>- The decline of orofecal and foodborne infections and changes in pathogens that stimulate the gut associated lymphoid tissue may be strong determinants of asthma and allergic rhinitis in developed countries.
**Microbiome: development**	Jain (2020)[13],#	- The first 1000 days of life may provide opportunity for microbial imprint of immunity, setting the standard for reactivity of the immune system for life:<br>- Caesarean-section babies have been shown to have higher risk for developing allergies and asthma compared to vaginally delivered babies. However, differences in the microbial community structure disappear after 6 Months of life with supplementary food introduction.<br>- Microbial imprint may change the programming of the development of immune cell subsets (e.g. via directing hematopoetic progenitors towards immune developmental pathways), or by changing their functionality.

		- Lack of (diverse) microbial exposures can lead to pathological imprinting that contributes to increased susceptibility to asthma, allergies, and chronic inflammatory conditions.
	Arishi et al. (2023)[78], #	Early feeding practices (breastfeeding and the introduction of solids) appear to be the strongest determinants of the infant oral microbiome:<br>- The diversity increases in infancy and early toddlerhood until it stabilises when early childhood is reached.<br>- Cessation of breastfeeding drives maturation of the infant gut microbiota, which might be true for oral microbiota as well.<br>- Solid food introduction might increase the complexity of the infant oral microbiome
**Allergy and microbiome (mucosal surfaces of skin, lung/nose, gut)**	Haspeslagh et al. (2018)[75], #	- Reference to alterations in the composition of the skin, nose, gut microbiome in association with atopic dermatitis, asthma or food allergy.<br>- The loss of sufficient microbial stimulation in western lifestyles may result in hypersensitive barrier tissues and the observed rise in type 2 allergies.<br>- The tolerance to allergens depends on microbial colonisation and immunostimulatory environments in early life.<br>- Through integration of allergens into barrier epithelial cells, a signal pathway is initiated where dendritic cells regulate or impede T cell responses via promoting either inflammatory or tolerogenic immunity.
	Feehley et al. (2019)[82], ¶	- Germ-free mice which were colonised with bacteria from faeces of healthy infants and subsequently sensitised with cow's milk protein beta-lactoglobulin (BLG), showed no anaphylactic response to BLG challenge compared to mice which were colonised with bacteria from faeces of cow's-milk allergic (CMA) infants which did.<br>- Healthy (n=31) and CMA (n=42) colonised mice also showed differences in transcriptome signatures in the ileal epithelium.<br>- Ileal bacteria *Anaerostipes caccae* protected against an allergic response to food.
	Cukrowaska et al. (2020)[19], #	- The most important environmental factors affecting microbiota formation in early life include C-section, use of antibiotics and infant feeding.<br>- Lower diversity of the gut microbiome is linked with the occurrence of allergies.<br>- *Bifidobacterium* species are important in microbiota modulation towards anti-allergic processes.
**Allergy and oral microbiome**	Dzidic et al. (2018)[37], †	- Children with allergic diseases (n=47) had significantly lower oral bacterial diversity at 7 years than healthy children (n=33). There was no difference in diversity at 3, 6, 12, and 24 months, but in relative abundance of specific bacterial species at all time points.<br>- Delivery mode, breastfeeding duration, antibiotic intake and maternal allergy did not show an influence on the microbiota in relation to allergy development in this study. However, most of the infants were exclusively breastfed until 3 months of age.

Article/Study Type: Observational studies (†) prospective, ‡) retrospective, §) cross-sectional); ¶) in vivo (animal) study; #) review; &) comment

*Disclaimer: The information presented in these columns is not guaranteed to be complete or accurate, and reflects solely the personal opinions of the authors
